# Heat Shock Protein 90 Positively Regulates Chikungunya Virus Replication by Stabilizing Viral Non-Structural Protein nsP2 during Infection

**DOI:** 10.1371/journal.pone.0100531

**Published:** 2014-06-24

**Authors:** Indrani Das, Itishree Basantray, Prabhudutta Mamidi, Tapas K. Nayak, Pratheek B. M., Subhasis Chattopadhyay, Soma Chattopadhyay

**Affiliations:** 1 Infectious Disease Biology, Institute of Life Sciences, Bhubaneswar, Odisha, India; 2 School of Biological Sciences, National Institute of Science Education & Research, Bhubaneswar, Odisha, India; Singapore Immunology Network, Agency for Science, Technology and Research (A*STAR), Singapore

## Abstract

**Background:**

The high morbidity and socio-economic loss associated with the recent massive global outbreak of Chikungunya virus (CHIKV) emphasize the need to understand the biology of the virus for developing effective antiviral therapies.

**Methods and Findings:**

In this study, an attempt was made to understand the molecular mechanism involved in Heat shock protein 90 (Hsp90) mediated regulation of CHIKV infection in mammalian cells using CHIKV prototype strain (S 27) and Indian outbreak strain of 2006 (DRDE-06). Our results showed that Hsp90 is required at a very early stage of viral replication and Hsp90 inhibitor Geldanamycin (GA) can abrogate new virus particle formation more effectively in the case of S 27 than that of DRDE-06. Further analysis revealed that CHIKV nsP2 protein level is specifically reduced by GA treatment as well as HSP90-siRNA transfection; however, viral RNA remains unaltered. Immunoprecipitation analysis showed that nsP2 interacts with Hsp90 during infection; however this interaction is reduced in the presence of GA. In addition, our analysis on Hsp90 associated PI3K/Akt/mTOR signaling pathway demonstrated that CHIKV infection stabilizes Raf1 and activates Hsp90 client protein Akt, which in turn phosphorylates mTOR. Subsequently, this phosphorylation leads to the activation of two important downstream effectors, S6K and 4EBP1, which may facilitate translation of viral as well as cellular mRNAs. Hence, the data suggests that CHIKV infection is regulated by Hsp90 associated Akt phosphorylation and DRDE-06 is more efficient than S 27 in enhancing the activation of host signaling molecules for its efficient replication and virus production.

**Conclusion:**

Hsp90 positively regulates Chikungunya virus replication by stabilizing CHIKV-nsP2 through its interaction during infection. The study highlights the possible molecular mechanism of GA mediated inhibition of CHIKV replication and differential effect of this drug on S 27 and DRDE-06, which will be informative for developing effective anti-CHIKV therapies in future.

## Introduction

Chikungunya virus (CHIKV), a mosquito borne arbovirus responsible for causing Chikungunya fever is transmitted mainly by *Aedes species* of mosquito. CHIKV belongs to Alphavirus genus and Togaviridae family [1], [2].The infection is characterized by high fever, nausea, rashes over the skin and polyarthralgia which is the hallmark symptom of CHIKV infection [2]–[5]. Although the virus was identified 50 years back but recent emergence of CHIKV as massive outbreaks from 2005 onwards in different parts of Indian Ocean, Asia and South East Asian continents emphasizes the urgency to study the virus extensively. CHIKV is an enveloped positive sense single stranded RNA virus and the 11.8 Kb long genomic RNA encodes four non-structural (nsP1–4) and five structural proteins (capsid, E1 and E2 glycoproteins, 6 k and E3) [3], [6], [7]. The four non-structural proteins are involved in viral replication and transcription. Considering the reports available in different Alphaviruses, it can be stated that nsP1 protein has methyl and guanyltransferase activity [8], nsP2 has helicase, NTPase and protease activities, nsP3 is known to be an accessory protein of nsP4 for RNA synthesis and nsP4 has the RNA dependent RNA polymerase activity [3].

Till date, our understanding of the involvement of cellular proteins for efficient viral infection and replication is incomplete. Hence, the identification of the cellular proteins and their role in replication and infection needs to be determined. It has been reported that viral infections induce cellular expression of stress response proteins like heat shock proteins (Hsps) [9]. Such induction of heat shock proteins have been reported for both the DNA and RNA viruses. However, the type of Hsp associated in a viral infection depends on the kind of pathogen and the nature of the host cells. [9], [10].The Hsps are known as important molecular chaperones that modulate different cellular processes to maintain cellular homeostasis [11]. Chaperones bind to misfolded or unfolded polypeptides to assist in their correct folding and assembly, regulate protein transport and translocation and facilitate misfolded polypeptides for degradation by the ubiquitin-proteasome system to maintain cell viability [11]–[14]. Although the assembly of cellular chaperones often increases with virus infection but it is still not clear whether this is a direct effect of infection or an indirect response to cellular stress induced by infection [9], [15], [16]. Moreover, any kind of stress or infection results in the induction of various Hsps like Hsp90, Hsp70, Hsp40 and several small Hsps [17], [18]. Hsp90 is considered as one of the highly expressed chaperone in cytoplasm [19].

Importance of Hsp90 in viral replication has similarly been reported in HCMV, Human immunodeficiency virus-1 (HIV-1), HCV, HEV, HSV-1, Vaccinia virus, HBV and Rotavirus [20]–[27]. Geldanamycin (GA), a potent Hsp90 inhibitor and its analogue 17-AAG as well as 17-DMAG, bind to the N-terminal ATP/ADP-binding pocket of Hsp90 with high affinity [28], [29]. As a result, Hsp90 is inactivated which leads to the destabilization and degradation of Hsp90 associated client proteins [30]–[32]. These client proteins like Raf1, Akt, Ksr1, Src are the components of various signal transduction pathways which are involved in cell proliferation, differentiation, growth arrest and apoptosis [33]–[36].

Recently it has been reported that Hsp90 inhibitor drugs, GA and two other drugs HS-10 and SNX-2112 can reduce CHIKV infection *in vitro* and *in vivo* [37]. Moreover, interactions between the Hsp90 protein and CHIKV nsP3 and nsP4 proteins have been identified [37]. This work supports the important role of Hsp90 during CHIKV infection, however, in depth understanding regarding the molecular mechanism of CHIKV mediated regulation of Hsp90 associated host cell response remains obscure and that opens up the possibility of Hsp90 for further investigation towards CHIKV biology, infection and replication.

In this study an attempt was made to understand the molecular mechanism involved in Hsp90 mediated regulation of CHIKV infection in mammalian cells using CHIKV prototype strain (S 27) and Indian outbreak strain of 2006 (DRDE-06) as we reported earlier that the 2006 Indian outbreak strain exhibits different pattern of infection in comparison to the prototype strain [38]. This was performed by using Hsp90 inhibitor, GA during viral infection and assessing its effect on viral replication and modulation of cellular proteins involved in Hsp90 associated signaling pathway.

## Materials and Methods

### Cells, Viruses, Antibodies and GA

Vero cells (African green monkey kidney epithelial cells), Chikungunya virus strains, S 27 and DRDE-06 were gifted by Dr. M. M. Parida, DRDE, Gwalior, India. Cells were maintained in Dulbecco's modified Eagle's medium (DMEM; PAN Biotech, Germany) supplemented with 5% Fetal bovine serum (FBS; PAN Biotech), Gentamycin, and Penicillin-Streptomycin (Sigma, USA). A monoclonal antibody of nsP2 [39] and polyclonal antibodies of nsP1, nsP2 [38], nsP3, nsP4 were developed by us (manuscript under preparation). Rabbit polyclonal antibodies to Raf1, Akt were purchased from Sigma (Sigma, USA); GSK3β, mTOR, S6K, 4EBP1 were purchased from Epitomics (Epitomics, USA); Ras, pmTOR, p70S6K, p4EBP1 were purchased from Cell Signalling (Cell Signalling Inc, USA). Hsp90 mouse monoclonal antibody was procured from BD(BD Biosciences, USA) and Hsp90 rabbit polyclonal antibody was procured from Cell Signalling (Cell Signalling Inc, USA) and pAkt, GAPDH antibodies were procured from Imgenex India (Imgenex India, India). Anti-mouse and anti-rabbit AP-conjugated secondary antibodies were purchased from Promega (Promega, USA). Hsp90 inhibitor, GA was purchased from Calbiochem (Calbiochem, USA).

### Cellular cytotoxicity determination

Vero cells were seeded in 96 well plates at a density of 3000 cells per well (Corning, USA) and after the cells had attained 90% confluency, they were treated with different concentrations of GA for 16 hours (h) at 37°C in 5% CO2. DMSO treated cells served as control for the experiment. Cellular cytotoxicity assay was performed according to the manufacturer's protocol. In brief, after 16 h of treatment with the drug, 10 µl of MTT reagent (5 mg/ml, Sigma, USA) was added to the wells followed by incubation for 3 h at 37°C. Later, the medium was removed carefully without disturbing the formazan crystals and 100 µl of DMSO was added (to dissolve the insoluble purple formazan product) followed by incubation at 37°C for 15 min. The absorbance of the suspension was measured at 540 nm using ELISA plate Reader. The percentages of metabolically active cells were compared with the percentage of control cells of the same culture plate. Cellular cytotoxicity was determined in duplicate and each experiment was repeated three times independently.

### Chikungunya virus infection

Vero cells with 90% confluency were grown in 35 mm or 60 mm cell culture dishes (according to the experimental requirements) in DMEM growth medium. The cells were infected with S 27 or DRDE-06 strains of CHIKV as described earlier [39], [40] with Multiplicity of Infection (MOI) 0.01, unless otherwise indicated. Samples were collected at different hours post infection (hpi) according to the assay. Infected cells were examined at 18 and 24 hpi with or without the drug (GA) for the detection of Cytopathic Effect (CPE).

### Western blot

Protein expression was examined by Western blot analysis according to the procedure described earlier [39], [40] In brief, Vero cells were harvested at different times post infection and then lysed using the RIPA buffer. Equal amount of protein (70 µg) was separated on 10% SDS-polyacrylamide gel and blotted on to nitrocellulose membrane. Protein expressions were checked with monoclonal nsP2 antibody (1∶3000 dilution) and the same was reprobed with GAPDH antibody (1∶15,000 dilution) to confirm the equal load of samples. Rabbit polyclonal antibodies to Raf1, Akt, GSK3β, mTOR, S6K, Ras, pmTOR, pS6K, p4EBP1 and Mouse monoclonal antibodies to Hsp90, pAkt were used at concentrations recommended by the manufacturer. In this study all Western blots were performed at least three times and the blots were scanned using Quantity One Software (Bio Rad, USA).

### Plaque assay

Vero cells were seeded on 6 well plates and were infected with viruses as mentioned above. The supernatants of infected cells (DMSO treated as well as GA treated) were collected as per the experimental requirements. Plaque assay was performed according to the procedure mentioned before [40]. The dishes were overlaid with methyl cellulose and maintained at 37°C. The cells were fixed once plaques were visible (4 to 5 days pi) and the viral plaque forming units were counted.

#### RT-PCR

The presence of CHIKV RNA in the infected cells was assessed by RT-PCR. Vero cells were infected with DRDE-06 virus and treated with 10, 50 and 100 µM of the drug for 14 h. Total cellular RNA was isolated using Trizol Reagent (Invitrogen, USA). RT was performed with 1 µg RNA by using the First Strand cDNA synthesis kit (Fermentas, USA) as per the manufacturer's instructions. In order to detect all viral cDNAs, each sample was subjected to PCR amplification using specific primers for CHIKV nsp1, nsp2, nsp3, nsp4 (manuscript under preparation), E1 and HSP90 [41], [42]. The RT-PCR products were subjected to 1.5% agarose gel electrophoresis and GAPDH served as internal amplification control.

### Immunoprecipitation

Confluent Vero cells grown in 100 mm dishes were infected with either CHIKV strains S 27 or DRDE-06 with MOI 0.1 as mentioned above and cells were harvested at 12 hpi. Cells were lysed with RIPA buffer and the clear supernatant was subjected to immunoprecipitation according to the procedure described previously [43]. In brief, the lysate was pre-cleared with normal rabbit or mouse immunoglobulin G and incubated with primary antibody (polyclonal antibody for nsP2 or monoclonal antibody for Hsp90) for 2 h at 4°C followed by incubation with protein G-agarose beads (GE Health care, USA). Finally, phosphate-buffered saline (PBS) and SDS-lysis buffer were added and samples were boiled for 10 min. Next, the immunoprecipitated proteins were separated on 10% SDS-Polyacrylamide gel and subjected to Western blot analysis by probing with either CHIKV-nsP2 monoclonal antibody or Hsp90 polyclonal antibody.

### Flow Cytometric Analysis of Annexin V

The Mock and CHIKV infected Vero cells in combination of GA treatment at different doses (50 µM and 100 µM) were harvested after trypsin EDTA treatment as mentioned in Material and Method section. For flow cytometric detection of apoptotic cells, Annexin V staining was carried out by using BD Annexin V Apoptosis Detection Kit I(BD Biosciences, USA) [44]according to manufacturer's protocol. Briefly, cells were detached by trypsin EDTA treatment. Cells were washed twice in ice cold PBS and then resuspended in 100 µl of 1X Annexin V binding buffer at a concentration of 1×10^6^ cells/ml. Then 2.5 µl of APC conjugated Annexin V was added per sample, gently vortexed and incubated for 15 min at RT (25°C) in the dark. After incubation, 400 µl of 1X Annexin V binding buffer was added to each tube and analyzed by BD FACS Caliburflow cytometry with CellQuest pro software (BD Biosciences, USA). A total of approximately 5×10^3^ cells were acquired for each sample.

### siRNA Transfection

Monolayers of Vero cells with 70% confluncey (2×10^5^ cells/well) in 24 well plates were transfected with different amount of siRNA (10, 30 and 60 pmol) corresponding to HSP90AA1 mRNA sequence (sense GGAGCUAAUCCCUGAAUAU and anti-sense AUAUUCAGGGAUUAGCUCCfrom Eurogentec, Belgium) or with siRNA negative control. Transfection was performed using Icafectin-442 (Eurogentec, Belgium) according to the manufacturer instructions. In brief, Vero cells (in serum free DMEM) were transfected using different amount of Icafectin according to siRNA quantity. The transfected cells were infected with either CHIKV strains S 27 or DRDE-06 with MOI1 24 hours post transfection (hpt). Eight hours post infection, the cells were harvested to measure the nsP2 protein level by Western blot analysis.

#### Statistical Analysis

The statistical analysis of the experimental data was performed by using either unpaired Student's t test using Graph Pad Prism 5.0 Software and presented as mean ± standard deviation of three independent experiments (n≥3). However, one-way ANOVA test was used for more than two values of the data. In all the tests, *p* value less than 0.05 was considered to be statistically significant.

## Results

### Hsp90 inhibitor, Geldanamycin inhibits viral replication

**Figure 1:**
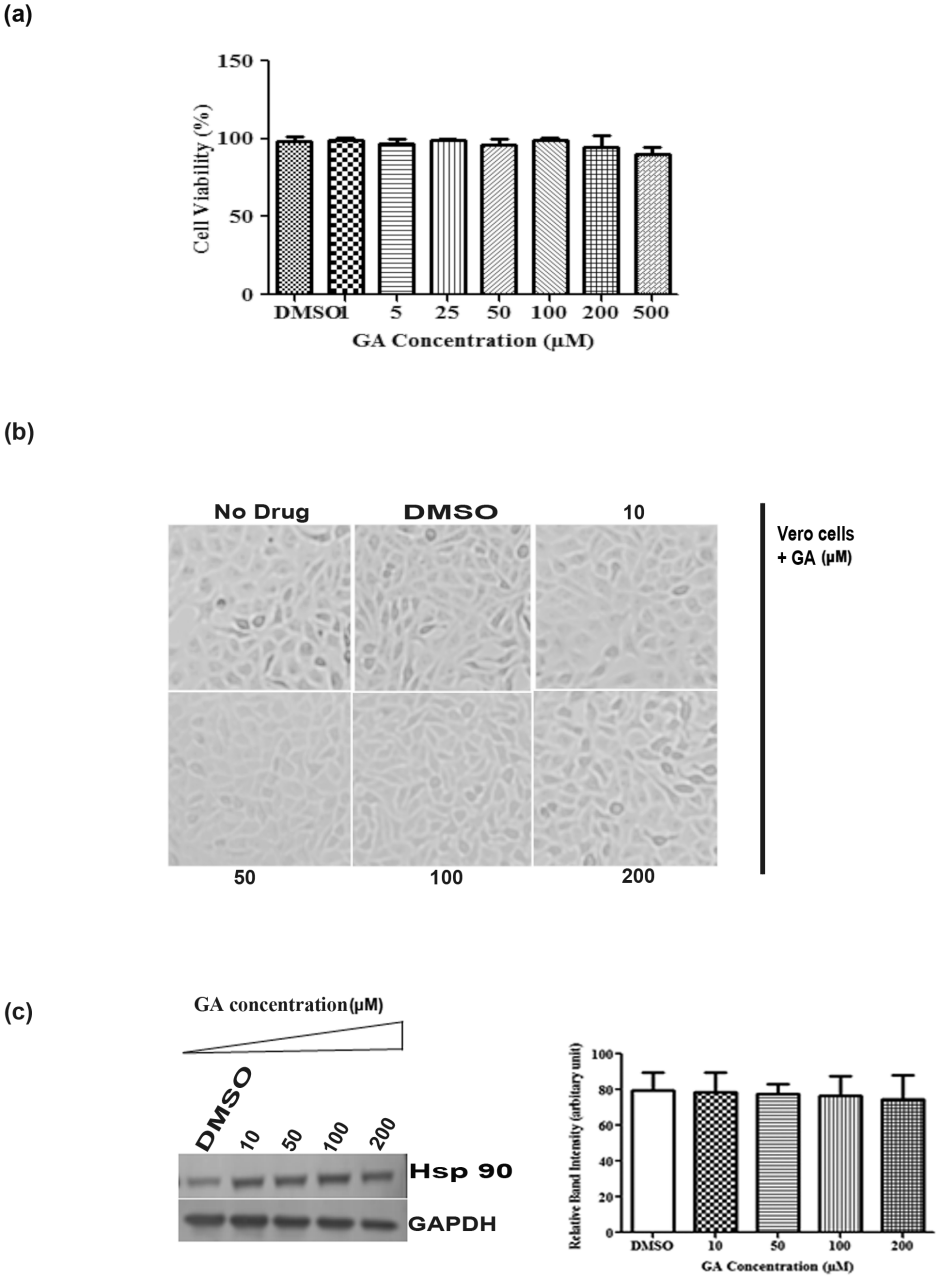
Determination of the cytotoxicity of Hsp90 inhibitor, Geldanamycin (GA). (a)Vero cells were treated with different concentrations of GA (1, 5, 25, 50, 100, 200, 500 µM) for 14 h and the cytotoxicity of the cells were determined by MTT assay. Cellular cytotoxicity was determined in duplicate and each experiment was repeated three times. (b) Cells were observed under microscope (Magnification -10X) for cytotoxicity with increasing concentration of the drug (10, 50, 100, 200 µM) on Vero cells at 14 h. (c) Vero cells treated with (10, 50, 100, 200 µM) of GA were harvested at 24 h, lysed and expression of Hsp90 was analysed in Western blot by probing with Hsp90 antibody. GAPDH served as the loading control. The changes in the band intensity was quantified by normalizing GAPDH and the relative band intensity has been shown as bar diagram in right panel (n = 3;*p*<0.05).

Since GA has been well known as a potent inhibitor of Hsp90, we intended to investigate its role in CHIKV infection in Vero cells. In order to determine the cytotoxicity of the drug GA, if any, Vero cells were treated with different concentrations of the drug (1, 5, 25, 50, 100, 200, 500 µM) for 16 h. Cytotoxicity was measured by MTT assay as mentioned in materials and methods. It was observed that 98% cells were viable with the lowest dose (1 µM) of the drug; however, 83% of the cells were viable with 500 µM concentration of the drug (Figure 1a). DMSO was used as reagent control. After drug treatment (10, 50, 100, 200 µM) CPE of the Vero cells were also observed under microscope and pictures were taken after 16 h of treatment. No CPE was observed when the cells were treated with 10, 50, 100 µM of the drug but little morphological changes were observed when 200 µM concentration of the drug was added as compared to the DMSO control (Figure 1b). Next, drug treated cells were harvested at 24 h, lysed and proteins were separated on 10% SDS-PAGE. After performing Western blot, the Hsp90 expression was assessed by probing the membrane with Hsp90 antibody.The Hsp90 expression was almost same after drug treatment as shown in Figure 1c and intensity of the bands were quantified in the right panel. Taken together, the result suggests that GA does not have cytotoxic effect on the Vero cells when treated with the above mentioned concentrations of the drug till 24 h.

**Figure 2:**
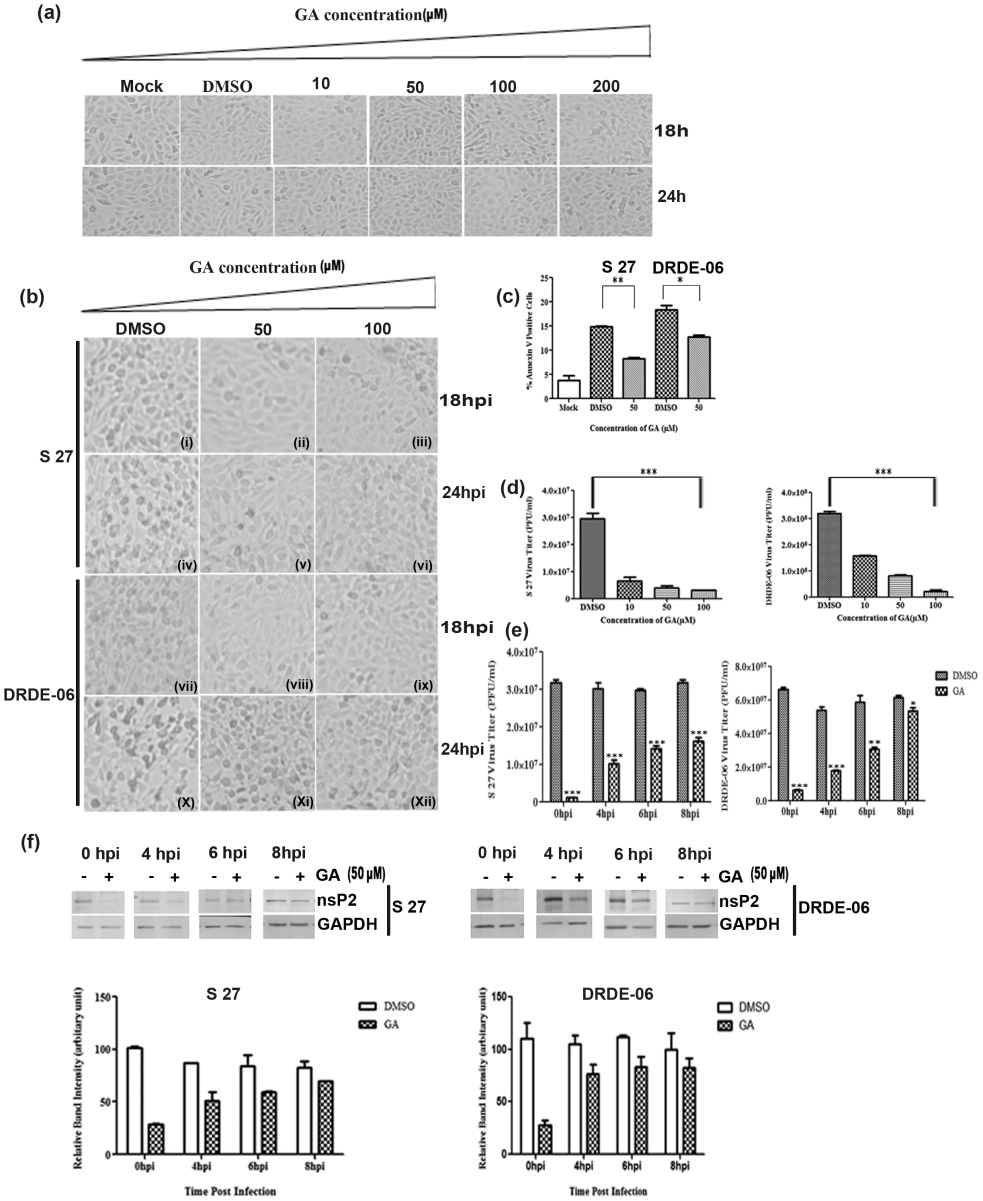
Hsp90 inhibitor GA inhibits CHIKV replication. (a) Vero cells were infected with CHIKV strains, either S 27 or DRDE-06 with MOI 0.01. Effect of GA (10, 50, 100, 200 µM) on (a) uninfected cells (b) in the progression of infection was determined by observing the CPE of the virus infected cells under microscope (Magnification -10X) at 18 and 24 hpi. (c) The mock and CHIKV (S 27 or DRDE-06) infected Vero cells in presence or absence of GA (50 µM) were analyzed for apoptosis by staining with Annexin V and estimating % positive cells for Annexin V. The graph depicts a representative experiment with triplicate values of mean ±SD (*p<0.05). (d) The supernatants of the virus infected and GA treated cells were collected at 14 hpi and viral titers were determined by plaque assay. The data represent the mean ±SD of three experiments (*p<0.01). (e) GA was added to the virus infected cells at 0, 4, 6, 8 hpi and supernatants were collected at 14 hpi and infectious progeny virus particle titre was determined by plaque assay. All the experiments were repeated three times and data of three independent experiments are represented as mean ±SD (*p<0.05). (f) The infected cells (as mentioned in 2e) were harvested and nsP2 protein level was determined by Western blot by probing with nsP2 monoclonal antibody. GAPDH served as the loading control. The fold change in the nsP2 protein level was quantified by normalizing GAPDH and the relative band intensity is shown as bar diagram in the lower panel (n = 3, *p*<0.01).

Next we were interested to determine the role of Hsp90 in CHIKV replication. The CHIKV prototype strain, S 27 and 2006 Indian outbreak strain DRDE-06 were selected for the study to elucidate the effect of GA in the progression of infection in Vero cells. Therefore, Vero cells were infected with CHIKV strains, S 27 or DRDE-06 with MOI 0.01. After1½ hrs of adsorption, GA (50 and 100 µM) was added to the infected cells, CPE was observed under microscope and pictures were taken at 18 and 24 hpi. As shown in Figure 2a, no characteristic morphological change was observed in uninfected Vero cells at 18 h or 24 h after GA treatment. Prominent CPE was observed in virus infected Vero cells with DMSO [Figure 2b (i, iv, vii, x)], while less CPE was observed after addition of GA [Figure 2b (ii, iii, v, vi, viii, ix, xi, xii)]. It was reported earlier that DRDE-06 replicates much faster than S 27 [38]. Similarly, in this study, GA mediated inhibition of viral infection was more in case of S 27 infected Vero cells [Figure 2b (ii, iii, v, vi)], in comparison to fast replicating DRDE-06 infected Vero cells [Figure 2b (viii, ix, xi, xii)]. Inhibition of replication was more pronounced when 100 µM of GA was used in comparison to 50 µM of the drug. It was reported earlier that CHIKV infection induces apoptosis in the host cell [45], thus for the quantitation of apoptosis of the virus infected cells in presence or absence of GA treatment, Annexin V staining was carried out to study the binding of the translocated membrane phospholipid phosphatidylserine of the apoptotic cells. It was observed that there was an induction of apoptosis during CHIKV infection (S 27 14.6±0.5 and DRDE-06 17.8±1.9) as compared to mock (4.6±2.6) (Figure 2c) as evident by higher number of Annexin V positive cells. However the level of apoptosis was reduced in presence of GA (S 27 8.4±1 and DRDE-06 12.6±0.8). This result confirms that GA reduces virus induced apoptosis and also supports the observation that GA treatment decreases CPE of infected cells as depicted in Figure 2b.

In order to assess the effect of GA in infective viral progeny formation, Vero cells were infected in similar fashion with the two virus strains as mentioned above (with or without drugs, GA-10, 50, 100 µM) and supernatants containing progeny virus particles were collected at 14 hpi. Plaque assay was performed to determine the progeny virus titre and it was observed that there was approximately 4.5 fold reduction (in presence of 10 µM GA) in the yield of infective progeny virus particle as compared to untreated cells in case of S 27. On the other hand, around two fold reduction (10 µM GA) in the yield of infective progeny virus particle formation was observed as compared to untreated cells in case of DRDE-06 (*p*<0.01). Thus, GA inhibited CHIKV replication in a dose dependent manner as observed in Figure 2d.

To understand whether functional Hsp90 is required during early or later stage of infection, Vero cells were infected with these two virus strains of CHIKV individually and GA (50 µM) was added to the media at 0 hpi, 4 hpi, 6 hpi and 8 hpi. After 14 hpi, cells and supernatants were harvested and viral titres were estimated. There was 96.55% inhibition in virus particle formation in case of S 27 and 90.33% reduction in case of DRDE-06 when GA was added at 0 hpi (Figure 2e). On the contrary, the inhibitory effect of the drug in viral particle production was reduced when drug was added at 4 hpi (Figure 2e) indicating that Hsp90 activity is required during early phase of viral infection. In consistent with the earlier observation, the inhibitory effect of GA was more pronounced in case of S 27 in comparison to DRDE-06. Moreover, the inhibitory effect of the drug was reduced gradually when the drug was added at 4, 6 or 8 hpi (*p*<0.01).

Next, CHIKV infected cells were lysed according to the protocol described above and Western blot analysis was performed to estimate the effect of GA on the synthesis of viral non-structural protein, nsP2. The blot was probed with nsP2 monoclonal antibody [38] and it was observed that the level of nsP2 was very less for S 27 as well as DRDE-06 when the cells were treated with the drug from 0 hpi (Figure 2f). However, the nsP2 protein was visible when the drug was added to the virus infected cells at 4, 6 or 8 hpi. The lower panels of Figure 2f show the quantitative measurement of nsP2 after normalizing GAPDH and error bars represent the S.D. of the data from three independent experiments. The result confirms the importance of Hsp90 function in CHIKV replication and indicates that the function of Hsp90 is required at the early stage of CHIKV replication.

### Hsp90 inhibitor reduces viral protein synthesis and virus release in presence of different amount of CHIKV

**Figure 3:**
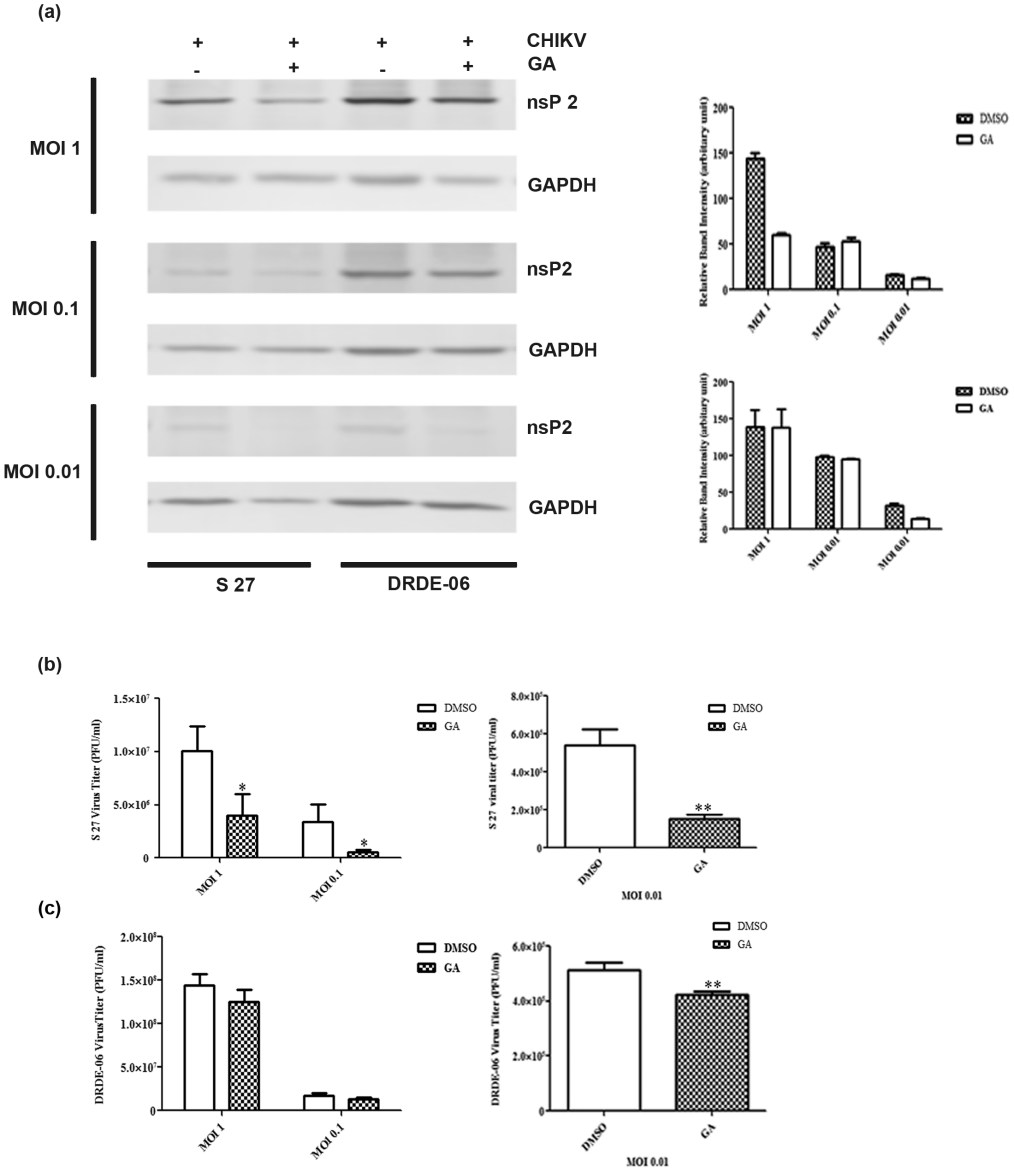
Effect of GA on virus replication with different MOIs of CHIKV. Vero cells were infected with different MOIs (1, 0.1, 0.01) of either S 27 or DRDE-06 virus and treated with 50 µM concentration of GA. (a) The cells were harvested at 8 hpi, lysed and Western blot analysis was performed by probing with nsP2 monoclonal antibody. GAPDH served as the loading control. The reduction in nsP2 level was quantified and the relative band intensity is shown as bar diagram in the right panel (n = 3, *p*<0.05) (b) and (c) The supernatants were collected at 8 hpi and infective progeny virus particle titre was determined by Plaque assay. Data of three independent experiments are represented as mean ±SD (*p<0.05).

In order to assess the magnitude of inhibition of Hsp90 inhibitor in different inoculum size of the two CHIKV strains, Vero cells were infected with different MOIs (1, 0.1, 0.01). The infected cells were harvested and supernatant containing new progeny viruses were collected at 8 hpi. At first the nsP2 protein synthesis was evaluated in Western blot and it was found that this protein was not detected when cells were infected with MOI 0.01 of S 27; a faint band was observed at MOI 0.1 and little more nsP2 synthesis was observed at MOI 1 (Figure 3a). Although, in case of DRDE-06 infection, the synthesis of nsP2 was reduced for all the MOIs as compared to DMSO, the inhibitory effect of GA was more in case of S 27 in comparison with the highly replicating epidemic potential virus strain DRDE-06 (Figure 3a). The right panels of Figure 3a show the quantitative measurement of nsP2 after normalizing GAPDH and error bars represent the S.D. of the data from three independent experiments. Next, the inhibitory effect was estimated by titrating the new progeny viruses by plaque assay. Figure 3b showed that the new virus particle release was reduced by 60.46% with MOI 1 (Figure 3b left panel) and 85.81% with MOI 0.01 in case of S 27 (Figure 3b right panel). Similarly, the viral titer was significantly reduced by 13.23% with MOI 1 (Figure 3c left panel) and 27.39% with MOI 0.01 in case of DRDE-06 (Figure 3b right panel). The results suggest that the viral protein level as well as new virus release both are affected to a great extent in lower MOI in case of S 27 and DRDE-06, however the drug is found to be less effective in the outbreak strain DRDE-06 (*p*<0.05).

### Hsp90 stabilizes nsP2 protein level during CHIKV infection

**Figure 4:**
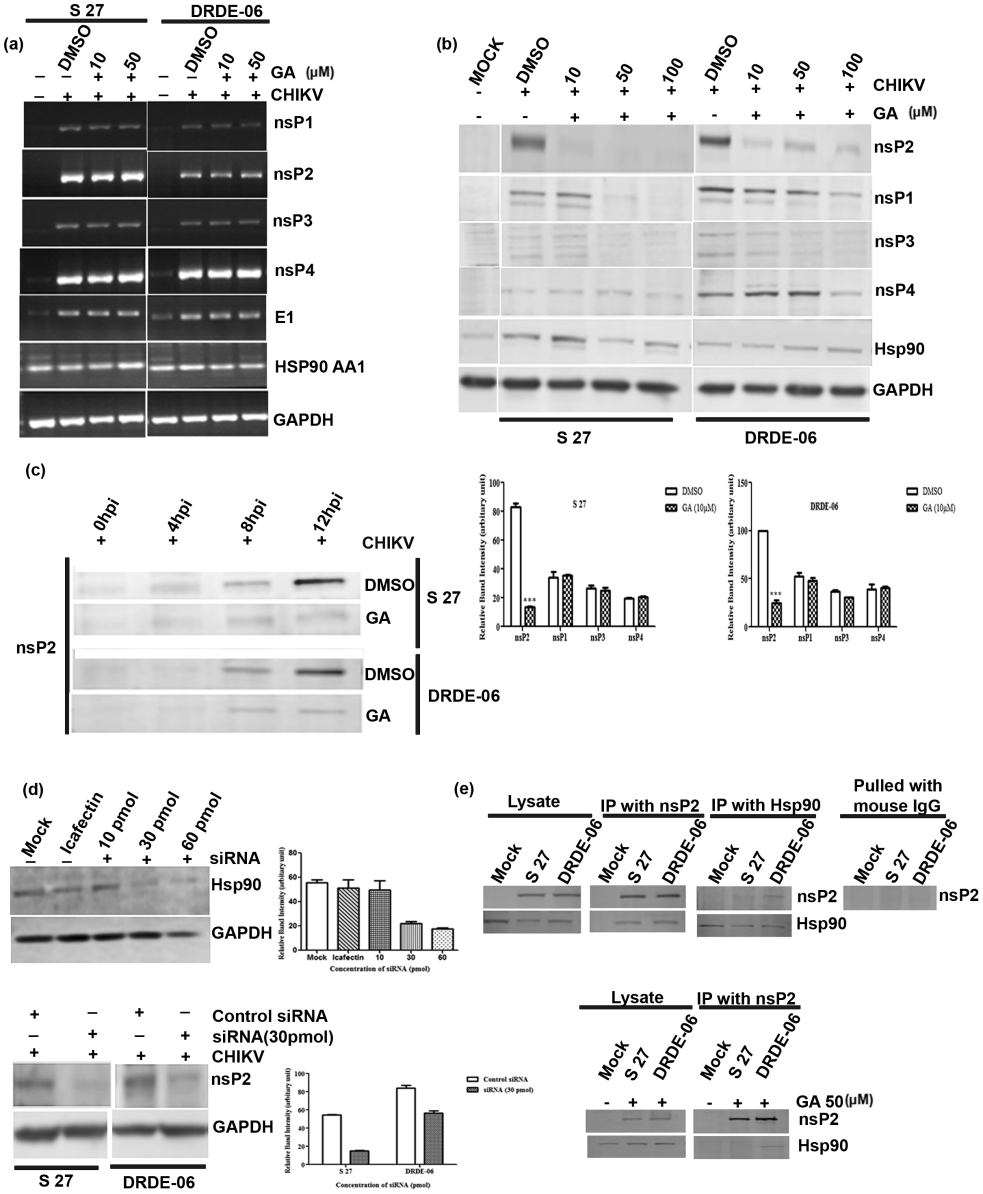
GA reduces nsP2 protein level during CHIKV infection. Vero cells were infected with either S 27 or DRDE-06 with MOI 0.01 of the virus. (a) Cells were treated with different doses (10 and 50 µM) of GA and the virus infected cells were harvested at 14 hpi and RT- PCR was performed to amplify nsP1, nsP2, nsP3, nsP4, Hsp90AA1 and GAPDH genes.(b) Virus infected cells were treated with 10, 50 and 100 µM doses of GA and harvested at 14 hpi. Western blot analysis was performed with cell lysates and probed with nsP1, nsP2, nsP3, nsP4 and Hsp90 antibody. GAPDH served as the loading control. The band intensities of nsP1-4 were measured for DMSO and 10 µM GA treated samples after normalizing GAPDH and error bars represent the S.D. of the data from three independent experiments (* *p*<0.01). (c) Vero cells were infected with either S 27 or DRDE-06 and treated with 50 µM GA. Cells were harvested at 0, 4, 8 and 12 hpi and probed with nsP2 monoclonal antibody. (d) Vero cells were mock transfected or transfected with 10, 30 and 60 pmol of HSP90AA1 gene siRNA. Hsp90 level was estimated in Western blot by probing with Hsp90 antibody (upper panel). After 24 hrt (30 pmol), cells were super infected with MOI1 of either S 27 or DRDE-06 and harvested at 8 hpi. nsP2 protein level was analysed by Western blot (lower panel). The changes in Hsp90 and nsP2 level were quantified by normalizing GAPDH and the relative band intensity is shown as bar diagram in the right panel (n = 3; *p*<0.05). (e) Vero cells were infected with either S 27 or DRDE-06 (MOI 0.1) virus in presence or absence of GA (50 µM). The cell lysates harvested at 10 and12 hpi for DRDE-06 and S 27 respectively, were co-immunoprecipitated with either polyclonal nsP2 or monoclonal Hsp90 antibodies. Western blot analysis was performed to check the interaction between nsP2 with Hsp90. The extreme right panel represents the negative control where normal mouse IgG was used to pull down the protein complex.

It was observed that the viral protein level was reduced in presence of Hsp90 inhibitor, thus we were interested to find out whether there was reduction in viral RNA content. In order to elucidate this, Vero cells were infected as mentioned above and cells were harvested at 14 hpi and RT-PCR was carried out. As shown in Figure 4a, it was observed that the RNA levels of viral proteins, nsP1, nsP2, nsP3, nsP4 and E1 were not affected after drug treatment. A non-specific band was observed in the mock sample for E1 gene, but the intensity was less than the actual virus infected sample. No significant change was observed in Hsp90 AA1 as well as in GAPDH (Figure 4a). GAPDH was used as RNA loading internal control. Next, the levels of the viral proteins were assessed by Western blot. In Figure 4b, the nsP2 protein level was remarkably reduced, whereas the other protein levels were reduced a little with increasing concentration of GA in case of both the viruses. This observation was supported by Figure 4b, lower panel where quantitative measurement of nsP1–4 band intensities for DMSO and 10 µM GA treated samples were represented as bar diagram (*p<0.01). In case of S 27, the nsP2 protein level was almost undetectable (left panel of Figure 4b) and Hsp90 protein level was unchanged in presence of the drug in case of both the viral infections. Viral proteins were detected only in infected lysates, but not in the mock sample as shown in Figure 4b. GAPDH was used as loading control. The same effect of the drug on the nsP2 protein level was observed in a time kinetics experiment when infected cells were harvested at 0, 4, 8 and 12 hpi in presence of GA (Figure 4c). Taken together, it can be suggested that GA suppresses mostly nsP2, but there are suppression of other proteins at a lower degree. Moreover, Hsp90 probably is involved in maintaining the stability of CHIKV-nsP2 and that is the reason why nsP2 level is reduced in presence of GA after CHIKV infection. The result again confirms that the drug seems to be less effective in case of the Indian outbreak strain of 2006 as compared to prototype strain S 27.

To confirm the importance of Hsp90 for CHIKV-nsP2 stability, siRNA was used to selectively silence the expression of HSP90AA1 gene in Vero cells. Twenty four hours after transfection of the siRNA, cells were analyzed by Western blot to measure Hsp90 level. It was observed that Hsp90 protein level was reduced by 60% as compared to negative siRNA transfected cells (Figure 4d). Next, the siRNA transfected Vero cells were infected with S 27 or DRDE-06 CHIKV strains and the cell lysates (8hpi) were analyzed by Western blot to assess the level of nsP2. Interestingly, it was observed that the nsP2 level was reduced by 72.73% after siRNA transfection as compared to the negative control (Figure 4d) The right panels of Figure 4d show the quantitative measurement of nsP2 after normalizing GAPDH and error bars represent the S.D. of the data from three independent experiments and the reduction in nsP2 level is statistically significant (*p*<0.05). Together, the result supports the previous observation that Hsp90 is required for CHIKV-nsP2 stabilization.

In order to understand the possible mechanism by which Hsp90 can stabilize CHIKV-nsP2, co-immunoprecipitation was performed with nsP2 as well as Hsp90 antibodies using virus infected cell lysates in presence or absence of GA. To our surprise, it was noticed that Hsp90 was pulled by nsP2 antibody and vice versa (Figure 4e) and interestingly this biochemical interaction between nsP2 and Hsp90 was reduced in presence of GA (Figure 4e lower panel). The nsP2 protein was not observed when the cell lysate was pulled with normal mouse IgG (Figure 4e extreme right panel) which indicates that nsP2 is specifically pulled by anti mouse Hsp90 antibody. The pull-down experiments indeed provide evidence for a strong interaction between nsP2 and functional Hsp90 which in turn sheds light on the mechanism of CHIKV-nsP2 stabilization during viral infection.

### Reduction in nsP2 level and virus titer when cells were serum starved

**Figure 5:**
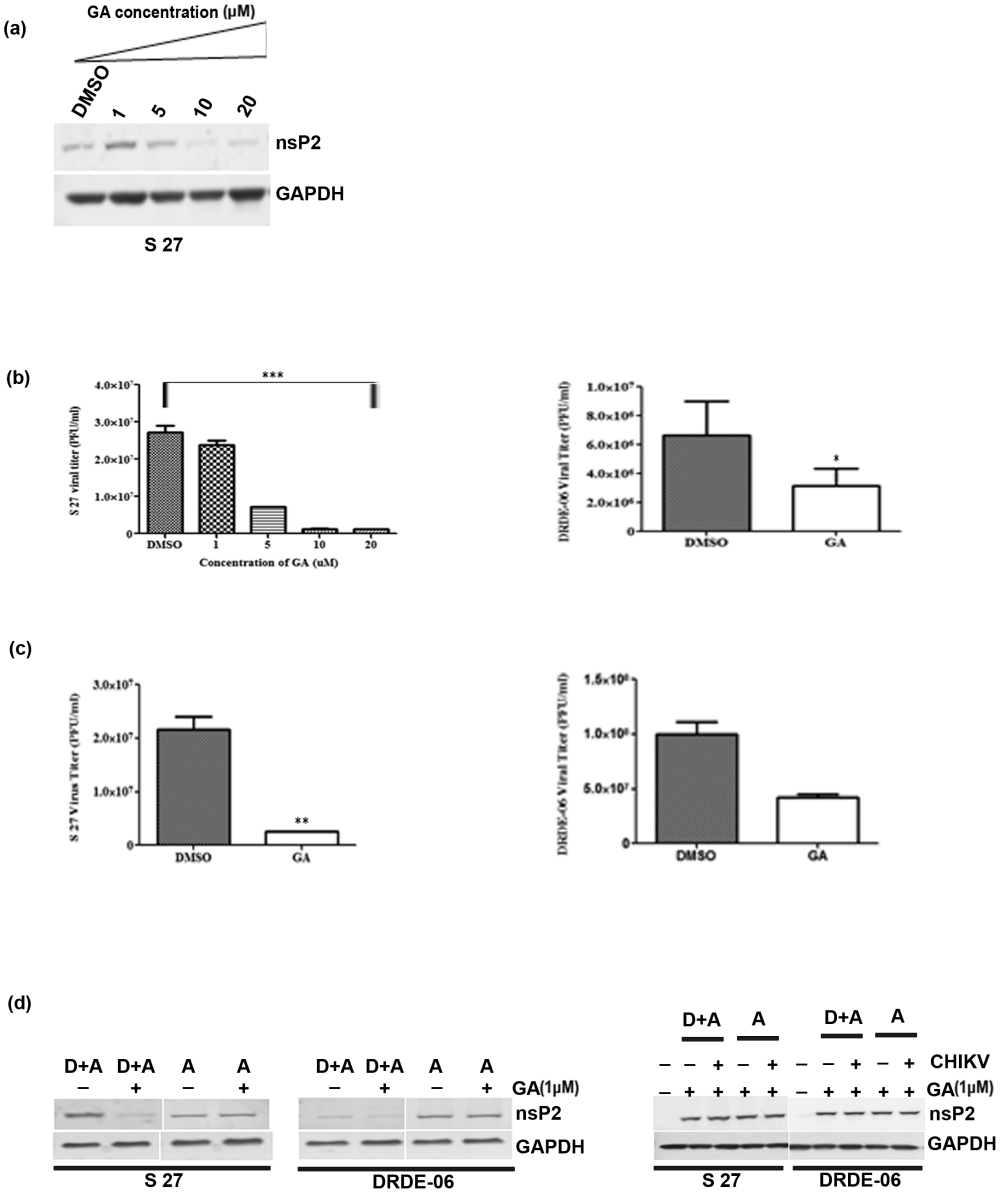
Reduction in nsP2 level and virus titer when cells were serum starved. Vero cells were serum starved for 48(a) Vero cells were infected with S 27 virus and treated with different concentrations of the drug (1, 5, 10, 20 µM) and cells were harvested at 14 hpi. Western blot was performed with cell lysates and probed with nsP2 antibody (b) Vero cells were infected either with S 27 or DRDE-06 and drug treated supernatants were collected at 14 hpi. Viral titre was determined by plaque assay. The data represents the mean ±SD with three independent experiments (*p<0.05). (c) Vero cells were infected with either S 27 or DRDE-06 and the drug (1 µM) was added during infection as well as after infection and the supernatants were collected at 14 hpi and plaque assay was performed to determine virus titre. Data of three independent experiments are represented as mean ±SD (*p<0.05). (d) The cells were harvested from the virus infected dishes as mentioned in 5(c) and Western blot was performed using the cell lysates collected from serum starved and nutrient rich condition (with and without 1 µM GA) and was probed for nsP2 and GAPDH antibody. A =  addition of GA after infection. D+A =  addition of drug during as well as after infection.

To reduce the basal level of the gene expression, 90% confluent Vero cells were maintained in absence of serum for 48 h and were infected with CHIKV prototype strain with MOI 0.01 according to the procedure mentioned earlier [20]. The cells and the supernatants containing the new progeny viruses were collected for detecting viral proteins and estimating viral titer. It was found that for S 27 virus infection, the nsP2 protein level was decreased in presence of 5 µM GA, whereas it was remarkably reduced in presence of 10 µM GA (Figure 5a). As shown in Figure 5b, left panel, 12.59% (27.26×10^6^ to 23.83×10^6^) reduction in viral titer was estimated in presence of 1 µM of GA for the S 27 virus infection, however, the production of new viral progeny particles was significantly reduced by 73.41% (27.26×10^6^ to 7.25×10^6^) in presence of 5 µM concentration of the drug. Moreover, the virus titer was reduced by 95.42% (27.26×10^6^ to 1.25×10^6^) in presence of 10 µM GA. Similarly, the effect of the drug in DRDE-06 virus infection was estimated. The virus titer was reduced by a greater extent, i.e. 57.47% (99.8×10^6^ to 42.45×10^6^) in presence of 1 µM of GA only (Figure 5b, right panel). The results indicate that the effect of the drug was more pronounced in the serum starvation situation instead of the normal serum supplemented condition and to our surprise the viral particle formation was inhibited to larger extent in case of DRDE-06 than S 27.

In order to understand the effect of Hsp90 inhibitor during the adsorption process, 1 µM concentration of the drug was added to the cells during one and half hours of adsorption and also after infection. As observed in Figure 5c, left panel, 88.46% (21.65×10^6^ to 2.5×10^6^) reduction was observed for S 27 when the drug was present during and after the infection. Similarly, in case of DRDE-06, 52.64% (27.26×10^6^ to 7.25×10^6^) reduction was observed when the drug was present during as well as after the infection (Figure 5c, right panel). When Western blot analysis was performed to check the effect of Hsp90 inhibitor on viral protein levels, there was a significant reduction in nsP2 level in case of both the viruses when GA was added during as well as after infection (Figure 5d left panel). However, surprisingly no significant change in nsP2 level was observed when the drug was added after infection in case of both the viruses. The level of nsP2 protein was assessed with the same concentration of GA in nutrient rich media and it was observed that there was no change in nsP2 level when 1 µM GA was added either after (A) CHIKV infection or during and after (D+A) viral infection (Figure 5d right panel). The data represents the mean ±SD with three independent experiments (*p*<0.05). Taken together, these results indicate that in the serum starved condition, the Hsp90 inhibitor interferes with the adsorption process of these two viruses and that finally lead to the reduction in viral protein synthesis and new progeny virus particle formation as well.

### Enhanced activation of Hsp client proteins after CHIKV infection

**Figure 6:**
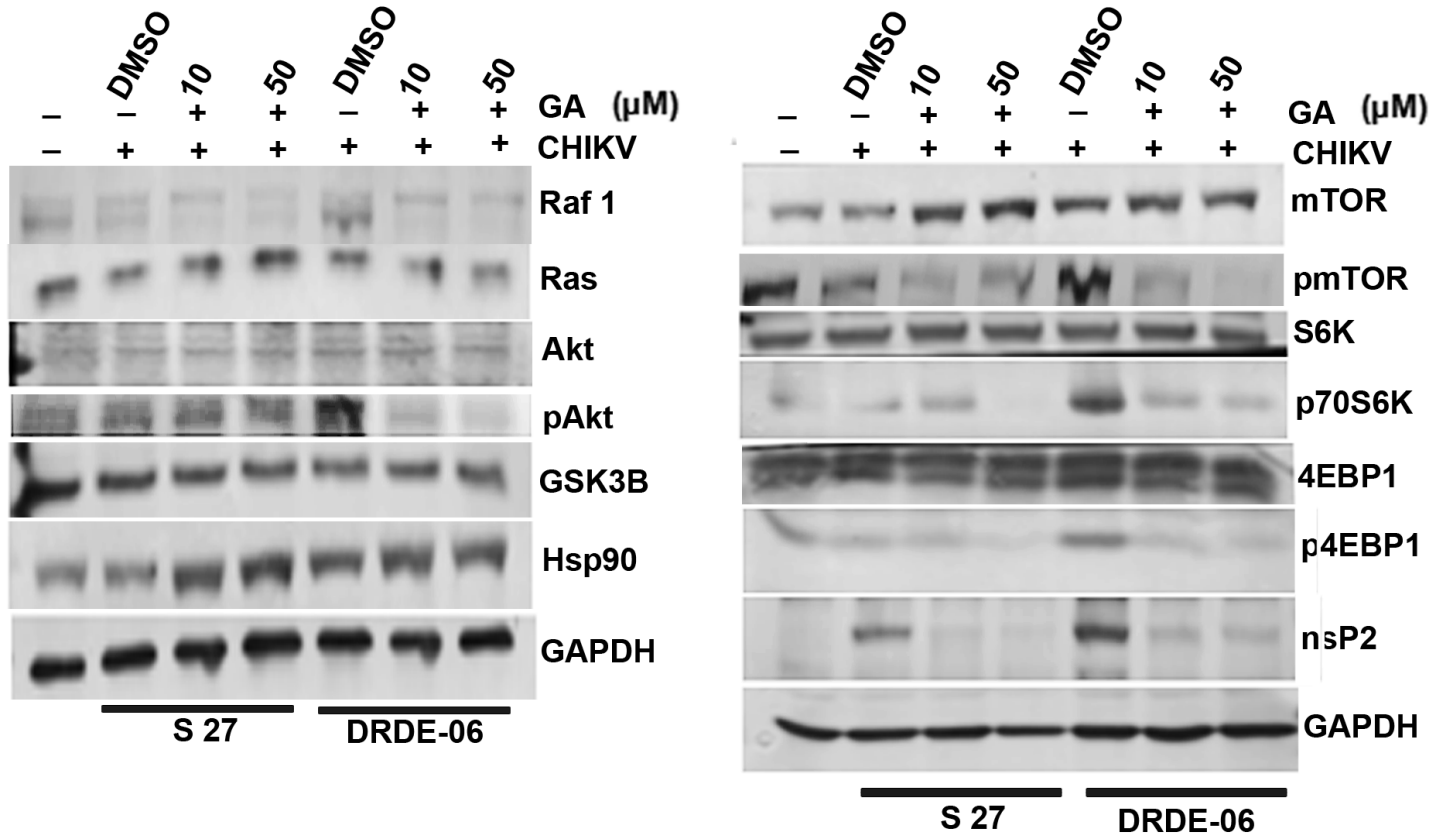
Enhanced activation of Hsp client proteins after CHIKV infection. Vero cells were infected with either S 27 or DRDE-06 with MOI 0.1 and treated with different doses of GA (10 and 50 µM) and the cells were harvested at 8hpi. Western blot was performed using cell lysates and probed with Hsp90, Raf1, Ras, Akt, pAkt, GSK3β, mTOR, pmTOR, S6K, p70S6K, 4EBP1, p4EBP1 and nsP2 antibodies. GAPDH was used as the loading control.

In order to understand the expression profile of Hsp90 associated client proteins during CHIKV infection, Vero cells were infected with either S 27 or DRDE-06 with MOI 0.1 and different concentrations of the drug (10 and 50 µM) were added to the cells after infection and the cells were harvested at 8 hpi. The level of viral protein, nsP2 was evaluated by Western blot analysis and it was found that nsP2 expression was significantly reduced at both the concentrations of the drug. However the reduction in nsP2 level was more evident in S 27 infection than DRDE-06. The expression of Hsp90 associated client proteins such as Raf1 (RNA polymerase activating factor 1) and Akt was checked and it was observed that the level of Raf1 was gradually reduced with the increasing concentration of GA (Figure 6 left panel). However, the level of expression of total Akt remained unaltered (Figure 6 left panel). Interestingly, during viral infection phosphorylation of Akt was found to be induced, whereas it was reduced upon addition of the drug (Figure 6 left panel).

To assess the effect of GA on Akt substrates such as regulator of the initiation of cap-dependent translation mTOR (mammalian target of Rapamycin), 4EBP1 (Eukaryotic initiation factor 4E binding protein 1), Ribosomal S6 kinase (S6K), Western blot analysis was performed using virus infected cell lysates (8 hpi) in the presence or absence of GA. In case of both the viral infections, S 27 as well as DRDE-06, there were induction in the phosphorylation status of various Akt substrates like mTOR, 4EBP1 and S6K than that of the mock cells (Figure 6 right panel). However, DRDE-06 virus infection resulted in an increase in phosphorylation of these Akt substrates more than that of S 27 infected cells, which was significantly reduced in presence of GA (Figure 6 right panel). This result indicates that DRDE-06 infection leads to enhanced translation of proteins required for viral replication and propagation in the host system than S 27 virus. Similar induction in host protein translation or activation of Hsp90 associated client proteins were observed for S 27 when infection was allowed to continue further (data not shown). However the basal level of expression of the proteins like Akt, GSK3β, Ras, mTOR, 4EBP1 and S6K remain unchanged during infection as well as in presence of GA (Figure 6).

## Discussion

In order to complete the replication of virus inside the host, several cellular proteins are required along with the viral non-structural proteins [46]. Cellular expression of Hsps are also known to be induced during viral infection of host cells [47], [48]. The high morbidity and socio-economic loss associated with the recent massive CHIKV outbreaks in different parts of the globe emphasize the need to understand the biology of the virus by identifying the host factors involved in CHIKV replication so that novel antiviral drugs can be developed to modulate this disease caused by the virus.

Recently it has been shown that Hsp90 is required for CHIKV replication and Hsp90 inhibitor, GA and two other derivatives of GA, HS-10 and SNX-2112 can reduce CHIKV infection. Moreover, the interactions between Hsp90 and CHIKV nsP3 and nsP4 proteins have been identified [37]. However, the molecular mechanism of this inhibition remains obscure. Hence, in the present study an attempt has been made to understand the molecular mechanism involved in Hsp90 mediated regulation of CHIKV infection in mammalian cells. We have shown earlier that the Indian outbreak strain of 2006 exhibits different pattern of infection as compared to the CHIKV prototype strain [38]. Accordingly, a comparative study has also been conducted to assess the effect of GA in case of prototype strain S 27 and the Indian outbreak strain DRDE-06.

Here, we confirm that Hsp90 inhibitor, GA reduces the CHIKV protein synthesis and viral particle formation for S 27 as well as DRDE-06. Our results showed that Hsp90 is required at very early stage of viral replication and Hsp90 inhibitor GA can abrogate new virus particle formation more effectively in case of prototype strain of CHIKV than that of the Indian outbreak strain of 2006. Further analysis revealed that GA treatment specifically suppresses the nsP2 protein in a large extent, whereas other viral proteins are suppressed at a lower degree. However, the level of viral RNA remains unaltered. Apart from GA, it was also observed that siRNA against Hsp90 can reduce the nsP2 protein level. Further, the pull down assay confirms the presence of interaction between active Hsp90 and nsP2 during infection and interestingly, this interaction was reduced in presence of GA. Surprisingly, the adsorption process of the virus was strikingly affected by the Hsp90 inhibitor in our study. Taken together, it appears that the failure of maintaining normal level of nsP2 may lead to the reduction of CHIKV progeny formation in absence of functional Hsp90. In addition, our analysis on Hsp90 associated PI3K/Akt/mTOR signaling pathway demonstrated that CHIKV infection stabilizes Raf1 and activates Hsp90 client protein Akt which in turn phosphorylates mTOR. Subsequently, this phosphorylation leads to the activation of two important downstream effectors, S6K and 4EBP1 which may facilitate translation of viral as well as cellular mRNAs. It was also observed that the 2006 Indian outbreak strain is more efficient in enhancing the activation of host signaling molecules in comparison to prototype strain for its efficient replication and virus production.

Association of Hsp90 in viral replication has been reported in HCMV, Human immunodeficiency virus-1 (HIV-1), HCV, HEV, HSV-1, Vaccinia virus, HBV, Influenza and Rotavirus [20]–[27], [49]–[54]. In addition, Hsp90 is known to interact with different signaling molecules like nuclear hormone receptors, tyrosine and serine/threonine kinases, cell cycle regulators and cell death regulators [55], [56]. Hsp90 also maintains the stability of the signaling molecules like Raf1, Akt which are involved in maintaining cell proliferation, differentiation, growth arrest and death [33], [57].Furthermore, viruses are known to manipulate PI3K-Akt-mTOR pathway for their survival [34], [58]–[63].

**Figure 7:**
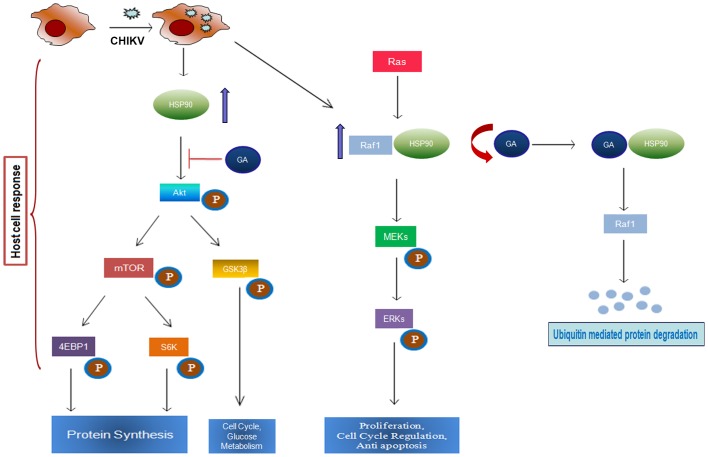
Schematic representation of the working model of the cellular signalling molecules in CHIKV infection. CHIKV infection results in the induction of Hsp90 associated client proteins like Raf1, Akt and various Akt substrates. GA treatment on CHIKV infected cells results in the degradation of Hsp90 associated client proteins which has been explained in the text.

In this study, we also tried to elucidate the role of Hsp90 in maintaining the stability of Hsp90 associated client proteins during CHIKV infection and also its role in modulating CHIKV induced intracellular signaling pathways. The modulation of PI3K/Akt/mTOR signaling pathway has been depicted schematically in Figure 7 considering our observation and available literature [34], [58]–[62]. In brief, upon CHIKV infection, Ras is activated which in turn targets Raf1. Hsp90 binds to Raf1 and helps in maintaining its stability [33], [57]. In addition, during CHIKV infection, the level of activated Akt (phosphorylated Akt) and activated mTOR (phosphorylated mTOR) are induced which leads to phosphorylation of 4EBP1 and S6K. The phosphorylation of S6K is involved in cell growth and proliferation, whereas phosphorylation of 4EBP1 leads to release of translational block [27], [62]. In contrary, the GA treatment to CHIKV infected cells results in non functional Hsp90 which ultimately fails to stabilize Hsp90 associated client proteins. This interferes with CHIKV replication and propagation in the host system. Hence, modulation of PI3K/Akt/mTOR pathway leads to enhanced translation of viral as well as host proteins which favours the virus.

In this study, it has been demonstrated that Hsp90 interacts with nsP2 and stabilizes its level during infection, however the actual mechanism of nsP2 stabilization needs further investigation. The nsP2 protein is a 799 amino acid long, multifunctional protein with key regulatory role for CHIKV replication [64]. The N-terminal part possess NTPase and RNA triphosphatase activity [65]. This domain also contains helicase activity, but the C-terminal part is essential for proper enzymatic activity of N-terminus.[64] Moreover the C-terminal part contains papain like protease activity [64] [66]. Alphavirus nsP2 localizes to the nucleus as well as cytoplasm during infection which indicates its involvement in cellular transcription shutoff and the CPE induction during CHIKV infection [67]–[69]. It was also observed that there are around 22 interacting host proteins of nsP2 which were identified by a high- throughput yeast two hybrid assay[4]. Further studies are required to find out the functional significance of those interactions. Surprisingly, Hsp90 was not identified as interacting protein in this study. As mentioned before, Rathore et al reported that Hsp90 is important for CHIKV replication and observed that nsP3 and nsP4 interact with Hsp90. In this report also nsP2 was not observed as an interacting protein of Hsp90 [37]. This might be because of the use of different CHIKV strains or cell types or different experimental set up. In this study, both the CHIKV strains (S 27 and DRDE-06) demonstrated interaction of nsP2 with Hsp90 during infection and it was also noticed that GA is less effective in reducing viral progeny formation or viral protein expression of DRDE-06 as compared to S 27. The possible explanation of this observation might be due to our previous observation of faster and enhanced replication of DRDE-06 than that of S 27 [38]. The detailed mutational analysis in our previous work also identified 19 mutations in the whole protein sequence of DRDE-06. Among those, two mutations are in nsP2 (S589N, A1328Y). Further investigation is required to address the role of mutations in the observed phenotypic differences and also to understand the mechanism in nsP2 stabilization.

It can be concluded that Hsp90 positively regulates Chikungunya virus replication by stabilizing CHIKV-nsP2 through its interaction during infection. Further, the study highlights the possible molecular mechanism of GA mediated inhibition of CHIKV replication and differential effect of this drug on S 27 and DRDE-06. Thus, the information will be useful for the development of effective anti-CHIKV therapies for controlling the viral infection in future.
