# Correction: Heat Shock Protein 90 Positively Regulates Chikungunya Virus Replication by Stabilizing Viral Non-Structural Protein nsP2 during Infection

**DOI:** 10.1371/journal.pone.0122906

**Published:** 2015-03-26

**Authors:** The PLOS ONE Staff

In Fig. 5d, the headings “CHIKV” and “GA (1μM)” are swapped in the first and second rows. The first row should be “GA (1μM)” and the second row should be “CHIKV.” Please see the corrected Fig. 5 here.

**Fig 5:**
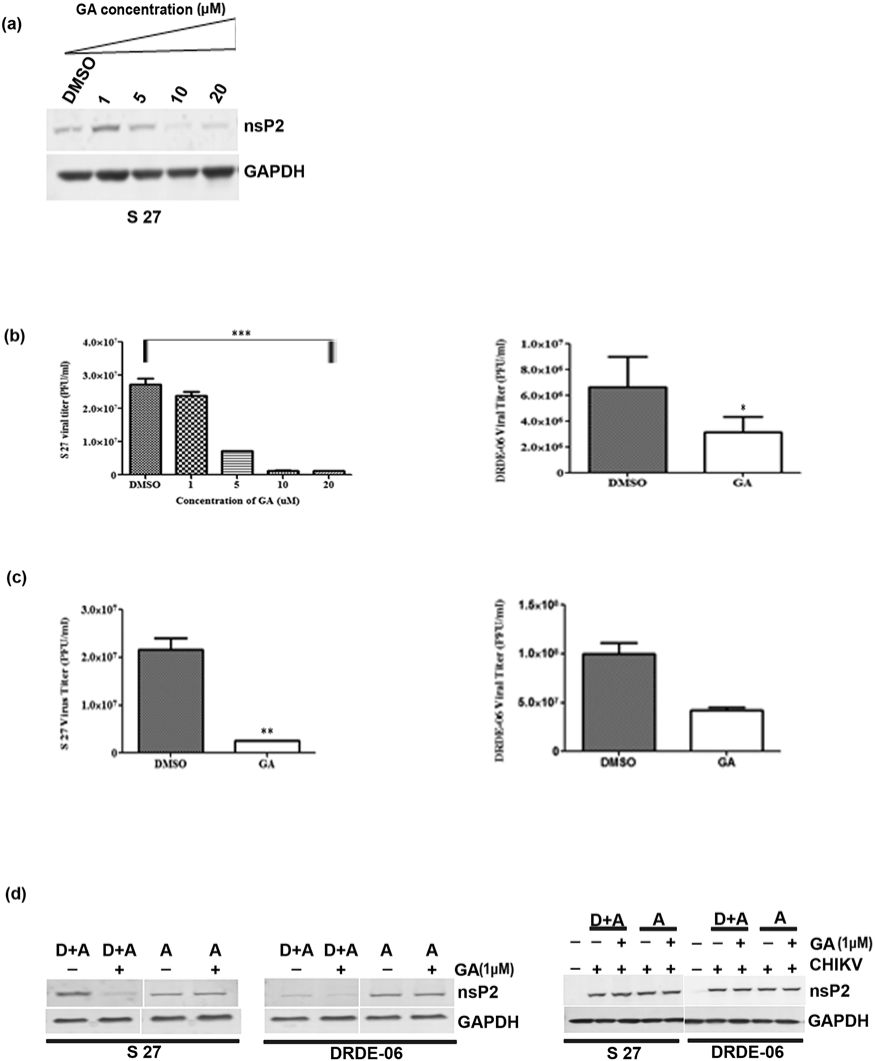
Reduction in nsP2 level and virus titer when cells were serum starved. Vero cells were serum starved for 48(a) Vero cells were infected with S 27 virus and treated with different concentrations of the drug (1, 5, 10, 20 μM) and cells were harvested at 14 hpi. Western blot was performed with cell lysates and probed with nsP2 antibody (b) Vero cells were infected either with S 27 or DRDE-06 and drug treated supernatants were collected at 14 hpi. Viral titre was determined by plaque assay. The data represents the mean ±SD with three independent experiments (*p<0.05). (c) Vero cells were infected with either S 27 or DRDE-06 and the drug (1 μM) was added during infection as well as after infection and the supernatants were collected at 14 hpi and plaque assay was performed to determine virus titre. Data of three independent experiments are represented as mean ±SD (*p<0.05). (d) The cells were harvested from the virus infected dishes as mentioned in 5(c) and Western blot was performed using the cell lysates collected from serum starved and nutrient rich condition (with and without 1 μM GA) and was probed for nsP2 and GAPDH antibody. A = addition of GA after infection. D+A = addition of drug during as well as after infection.
